# Sulfoxaflor Residues in Pollen and Nectar of Cotton Applied through Drip Irrigation and Their Potential Exposure to *Apis mellifera* L.

**DOI:** 10.3390/insects11020114

**Published:** 2020-02-10

**Authors:** Hui Jiang, Jianjun Chen, Chen Zhao, Yongqing Tian, Zhixiang Zhang, Hanhong Xu

**Affiliations:** 1 State Key Laboratory for Conservation and Utilization of Subtropical Agro-Bioresources, South China Agricultural University, Guangzhou 510642, China; jianghuizukai@stu.scau.edu.cn (H.J.); zhaoc@scau.edu.cn (C.Z.); tyq2006@scau.edu.cn (Y.T.); 2 Key Laboratory of Natural Pesticide and Chemical Biology, South China Agricultural University, Guangzhou 510642, China; 3 Department of Environmental Horticulture and Mid-Florida Research and Education Center, Institute of Food and Agricultural Sciences, University of Florida, Apopka, FL 32703, USA; jjchen@ufl.edu

**Keywords:** *Aphis gossypii*, *Apis mellifera*, drip irrigation, sulfoxaflor, residue level

## Abstract

Systemic insecticides have been applied through drip irrigation for controlling crop pests, but few studies have addressed potential negative effects of the application on non-target organisms. In this study, the safety of sulfoxaflor applied at 450 or 700 g a.i. ha^−1^ through drip irrigation at different times before flowering or during flowering to honey bee (*Apis mellifera* L.) was studied in 2016–2017 in a cotton production field in Xinjiang, China. Results showed that sulfoxaflor residues in pollen and nectar of cotton treated with sulfoxaflor at 450 g a.i. ha^−1^ before and during flowering through drip irrigation were either undetectable or no more than 17 μg·kg^−1^. Application of sulfoxaflor at 700 g a.i. ha^−1^ before flowering resulted in ≤ 14.2 μg·kg^−1^ of sulfoxaflor in pollen and < 0.68 μg·kg^−1^ in nectar. Sulfoxaflor applied at this higher rate during flowering had the highest residue, up to 39.2 μg·kg^−1^ in pollen and 13.8 μg·kg^−1^ in nectar. Risk assessments by contact exposure and dietary exposure showed that drip application of sulfoxaflor at the two rates before or during flowering posed little risk to honey bees. Thus, drip application of sulfoxaflor could represent an environmentally benign method for controlling cotton aphid.

## 1. Introduction

Drip application of chemicals or chemigation is a method of delivering insecticides to plant root zones for the control of crop pests [1,2,3,4]. Chemigation has numerous advantages, including uniform distribution of insecticides and reduction in application times, manpower, and cost [3,5,6,7]. Increasing reports have shown that insecticides applied via drip irrigation exhibited higher efficacy against crop pests than the conventional foliar spray method [2,3,4,6,8,9,10].

Xinjiang is the world’s most important cotton-producing region, accounting for 10% of the annual global cotton lint production and about 50% of the cotton yield in China [11]. Cotton production in Xinjiang is primarily irrigated through a drip system [12,13]. Cotton farming has served as an engine of economic growth and provided income to millions of farmers in cotton production regions [14]. However, cotton aphid (*Aphis gossypii* Glover) is a major recurrent pest in cotton plants and a notorious problem significantly affecting cotton lint yield and quality.

Foliar spraying of neonicotinoids, such as imidacloprid, thiamethoxam, and clothianidin, is a common method used for the control of aphid in Xinjiang. However, increasing negative impacts on important pollinators like bees had led to the ban of pesticide applications in open fields. Bees are the most important group of pollinators worldwide, 35% of the world food crop production depends on pollinators [15,16]. Sulfoxaflor is a novel sulfoximine insecticide, which exhibits high degree efficacy against a wide range of sap-feeding insect pests, including many that are resistant to neonicotinoids [17,18,19]. A recent report showed that drip irrigation of sulfoxaflor was more effective in the control of cotton aphid than conventional foliar spray [9]. Although sulfoxaflor and neonicotinoids are chemically distinct, they share a common biological mode of action [20]. This raises major concerns about the potential effects of sulfoxaflor on non-target species, particularly on honey bees. Some reports showed that sulfoxaflor had high toxicity to honey bees, and its LD_50_ (median lethal dose) was lower than 0.6 μg a.i./bee [21,22,23]. According to the experimental guideline for environmental safety evaluation of chemical pesticides, a pesticide is classified highly toxic to honeybees if the LD_50_ is < 2 μg a.i./bee [24]. A recent study reported that sulfoxaflor applied via foliar spray had severe sub-lethal effects on bumblebee (*Bombus terrestris* L.) colonies [23]. However, no one has reported the residue level of sulfoxaflor applied via drip irrigation in cotton flowers and its risk to honey bee (*Apis mellifera* L.) in an open field. Therefore, there is an urgent need to pre-emptively evaluate the potential effects of sulfoxaflor applied through drip irrigation on *A. mellifera* in cotton production field; such effects are rarely detected by standard ecotoxicological assessments but can have major impacts at larger ecological scales [25,26,27].

The present study was intended to determine the residue levels of sulfoxaflor applied through drip irrigation in cotton flowers in a cotton production field in Xinjiang. Sulfoxaflor at two rates were applied via drip irrigation at different times before and during flowering, and sulfoxaflor residues in pollen and nectar were tested. Additionally, the potential risk of the residue to honey bee was evaluated. Our results showed that the drip application of sulfoxaflor at the two rates posed little risk to honey bee during cotton production in Xinjiang.

## 2. Materials and Methods

### 2.1. Chemicals and Reagents

Sulfoxaflor of certified reference standard (98% purity, CAS number 946578-00-3) was bought from Shanghai Mingbo Biotechnology Co., Ltd (Shanghai, China). Sulfoxaflor 50% water dispersing granule (WDG) was provided by Dow AgroSciences (Zionsville, IN, USA). HPLC-grade acetonitrile, AR-grade acetonitrile, and sodium chloride were purchased from Tianjin Fuchen Chemical Reagent Factory (Tianjin, China). PSA and C_18_ were purchased from Sigma Aldrich (Steinheim, Germany). Ultrapure water was obtained using Millipore Milli-RO plus and Milli-Q systems (Bedford, MA, USA).

### 2.2. Experiment Design

The field experiments were conducted at Bole County, Xinjiang Uygur Autonomous Region, northwest China (44°20′–45°23′ N and 79°53′–83°53′ E) in 2016 and 2017, respectively. On 5 April 2016 and 24 April 2017, cotton seed (Xinliuzao-42) was sown in a field installed with a drip irrigation system under the film. The experimental field site was similar to our previously reported site [9].

Experiments were initiated on 3 June 2016 and 10 June 2017, and the application rate of sulfoxaflor was 450 g a.i. ha^−1^ and 700 g a.i. ha^−1^. The application rates were chosen based on previous test results of sulfoxaflor (drip irrigation: sulfoxaflor, 700 g a.i. ha^−1^) in a cotton field in Xinjiang [9]. The detailed calendar of sulfoxaflor treatments is presented Table 1. In both years, sulfoxaflor was applied one time during the entire experiment. Four application times with 450 g a.i. ha^−1^ or 700 g a.i. ha^−1^, plus controls resulted in a total of 12 treatments. The experiments were arranged as a completely randomized block design with 3 replicates. Thus, a total of 36 plots were prepared. Each block was 2400 m^2^ encompassing 12 plots, 200 m^2^ each, which was separated by a 4 m buffer zone. Each plot had 3200 to 3500 plants. Drip applications of sulfoxaflor at 450 g a.i. ha^−1^ or 700 g a.i. ha^−1^ following the days indicated in Table 1 based on the procedures previously described [9]. Drip controls were the same irrigation regime without any chemicals. For application during flowering, flowers were covered by wax-coated paper bags to prevent pollinator visits.

### 2.3. Sample Collection

The cultivar flowering lasted about one month (2016: from 3 July to 4 August; 2017: from 10 July to 11 August). The days for sampling pollen and nectar were as follows:

Sulfoxaflor applied 30 days before flowering: In 2016 and 2017, pollen and nectar samples were collected on day 35, 40, 45, and 50 after sulfoxaflor application, respectively.

Sulfoxaflor applied 20 days before flowering: In 2016 and 2017, pollen and nectar samples were collected on day 25, 30, 35, and 40 after sulfoxaflor application, respectively.

Sulfoxaflor applied 10 days before flowering: In 2016 and 2017, pollen and nectar samples were collected on day 15, 20, 25, and 30 after sulfoxaflor application, respectively.

Sulfoxaflor applied during flowering: In 2016 and 2017, pollen and nectar samples were collected at 2 h and on day 1, 3, 5, 7, 15, and 20 after sulfoxaflor application, respectively.

Open flowers were picked from plants and placed in bags. The bags were immediately placed in iceboxes and brought to the laboratory for extracting pollen and nectar based on the methods described by Dively and Kamel [28]. All operations were completed on ice. Pollen and nectar samples were frozen immediately and transported to the South China Agricultural University Pesticide Analytical Laboratory and placed in a −20 °C freezer until extraction.

### 2.4. Sample Preparation

Two grams of pollen or 1 mL of nectar samples were placed in a 10 mL conical centrifuge tube with 3.0 mL AR-grade acetonitrile and 0.5 mL water. All tubes were sonicated for 30 min, and 0.5 g sodium chloride was added, and vortexed mixed for 2 min. All tubes were centrifuged for 5 min at 6000 rpm, and acetonitrile extract (supernatant) was transferred into a 5.0 mL plastic centrifuge tube containing PSA (0.1 g) and C_18_ (0.1 g) and centrifuged at 6000 rpm for 5 min. The supernatants were evaporated to dryness in a water bath under a stream of N^2^ at 40 °C. The dried residue was then reconstituted in 2 mL of acetonitrile and filtered through 0.22 μm syringe filter (Nylon) into glass auto-sampler vials for LC-MS analysis.

### 2.5. Instrumentation and Condition

Prepared samples were analyzed by an Agilent UPLC-MS/MS (Infinity ultraperformance liquid chromatograph, Agilent, Beijing, China) with an Eclipse plus C_18_ column (50 mm × 2.1 mm, i.d. 1.8 μm particle size). The mobile phase consisted of 0.01% formic acid in water (solvent A) and acetonitrile (solvent B) applied at a flow rate of 0.2 mL/min under the following gradient conditions: (1) 0.05 min (A-B, 95:5, *v/v*); (2) 2 min (A-B, 5:95, *v/v*); (3) 5.5 min (A-B, 95:5, *v/v*); (4) 8 min (A-B, 95:5, *v/v*). Injection volume and column temperature were set at 5 μL and 30 °C. The mass spectrometer was operated with ESI source in the positive ionization mode, and sulfoxaflor was detected by multiple reaction monitoring (MRM) with 1 precursor ion and 2 product ions. Optimized MRM parameters of sulfoxaflor were as follows: Qualifying ion pairs were 278.1/174.0 *m/z* and 278.1/153.9 *m/z*, and the quantifying ion pair was 278.1/174.0 *m/z*; collision energy was 12 V and 20 V, declustering potential was 100 V. The LOQ (S/N = 10) and LOD (S/N = 3) were 3.87 μg·kg^−1^, 1.16 μg·kg^−1^ in pollen and 2.26 μg·kg^−1^, 0.68 μg·kg^−1^ in nectar, respectively. The mean recoveries of sulfoxaflor in pollen and nectar were within 85.67–92.33% and 83.00–96.18%, respectively. Additionally, the RSDs ranged within 2.46–3.06% and 1.17–4.04%, respectively.

### 2.6. Risk Assessment

To understand the risk of sulfoxaflor posed to *A. mellifera*, the risk assessment was estimated according to the flower hazard quotient (FHQ_do_) value [29]. The FHQ_do_ value was calculated from the predicted exposure concentration (PEC) in pollen and nectar multiplied by the maximum contact level (MCL)] and the acute contact LD_50_ for adult bees. According to the reference, the maximum contact level of honey bees was 1 g of contaminated flowers per day [29,30]. The contact LD_50_ was 0.585 μg a.i. bee^−1^ [22]. When the FHQ_do_ value was lower than 0.1, the risk was acceptable, while the value between 0.1 and 1 indicated moderate risk, and the value greater than 1 was considered to be an unacceptable risk.

The above method was suitable for contact exposure, but it may not be appropriate to assess risk by chronic dietary exposure because the bees constantly consume pollen, nectar, and honey. If the residues ingested remained in the body of honey bees, a LD_50_ could be reached after some time; meanwhile, compounds should have some elimination and metabolism [31], thus the cumulative residue amounts estimated by the above way could represent the worse-case scenario. Therefore, a further assessment of the dietary risk of sulfoxaflor was performed using the fixed-dose approach, where the estimated time to reach the oral LD_50_ value was compared with the actual lifespan of 3 types of honeybee (worker larvae, nurses, and foragers) [30]. The oral LD_50_ was 0.187 μg a.i. bee^−1^ [22]. When the time was shorter than the lifespan, it represented a serious risk [30]. The time reach to oral LD_50_ was calculated as follow:

(1) $$T50_{\;}(\mathrm{days})\;=\;\frac{\left(\mathrm{oral}\;\mathrm{LD}50\left(\mu g/\mathrm{bee}\right)\right)}{\mathrm{Daily}\;\mathrm{dose}\;\left(\mu g\right)}$$

The consumption rates of three types of *A. mellifera*. was referred to as the references [30,32]. Since nectar was to be dehydrated to concentrate the sugar to honey, we estimated daily consumption rates on the basis of total sugar intake (mg). According to the reference [33,34], nectar of cotton flowers contains between 17.9%–36.5% of sugar and honey contains, on average 80% of sugar. In the calculation process, we firstly converted honey to sugar and then sugar to nectar.

### 2.7. Statistical Analyses

All data were subjected to analysis of variance (ANOVA) using SPSS software (version 15.0; SPSS Inc., Chicago, IL, USA). When significance occurred, means were separated by Tukey’s HSD test (*p* < 0.05). The data of sulfoxaflor concentrations were presented as the mean ± standard errors with 3 replications.

## 3. Results

### 3.1. Sulfoxaflor Concentration in Pollen and Nectar

Sulfoxaflor concentrations in pollen and nectar of cotton plants produced in 2016 are presented in Table 2. Results showed that the levels of sulfoxaflor residue differed in pollen and nectar and also varied by application times and doses. Sulfoxaflor was not detectable in pollen and nectar when it was applied 30 days before flowering. When sulfoxaflor was applied 20 days before flowering at 700 g a.i. ha^−1^, it was detected in pollen only (< 9 μg·kg^−1^) 25 days after the application. When it was applied 10 days before flowering, sulfoxaflor concentrations up to 14.2 μg·kg^−1^ were detected in pollen of plants after 15 and 20 days of application. Application of the low dose during flowering, sulfoxaflor residues was detected in pollen only ranging from 7.7 to 17 μg·kg^−1^. Application of the high dose during flowering, sulfoxaflor residue varied from 11.7 to 39.2 μg·kg^−1^ in pollen and 6.6 to 13.8 μg·kg^−1^ in nectar depending on the days of sampling. Sulfoxaflor concentrations in pollen of plants treated with 700 g a.i. ha^−1^ during the flowering stage from day 5 to day 7 were significantly higher than the other treatments (*F =* 11.40, *df =* 13, 28, *p <* 0.001), but there were no significant differences within the other treatments.

Residue concentrations of sulfoxaflor in pollen and nectar of plants treated with two doses of sulfoxaflor at different times in 2017 were similar to those of 2016, which is presented in Table 3. Sulfoxaflor concentrations were ≤ 13.5 μg·kg^−1^ in pollen and below the detection limit in nectar when sulfoxaflor was applied before flowering. During flowering, drip application of two different doses of sulfoxaflor resulted in 8.9–34.6 μg·kg^−1^ in pollen and 7.6–10.8 μg·kg^−1^ in nectar. By comparison, sulfoxaflor concentrations in pollen of plants after 5 and 7 days of treatment with the high dose during the flowering period were significantly higher than the other treatments (*F =* 16.71, *df =* 12, 26, *p <* 0.001); there were no significant differences within the other treatments.

### 3.2. Potential Risk Assessment to A. Mellifera

The contact exposure of sulfoxaflor to honey bees in 2016 and 2017 was estimated by the flower hazard quotient (FHQ_do_) [29]. The results showed that FHQ_do_ values were lower than 0.1 for the two doses of sulfoxaflor applied either before or during flowering (Table 4; Table 5). The results showed that drip application of the two different doses of sulfoxaflor before or during flowering had little risks by contact exposure to honey bees.

The dietary exposure of sulfoxaflor was estimated by the T_50_ for representative three honey bee types. The results showed that all T_50_ values were longer than those lifespan of three different types of bees (Table 6 and Table 7) [30]. The results indicated that drip application of sulfoxaflor at the mentioned two rates should have no dietary exposure risks to three different types of honey bees.

## 4. Discussion

Cotton production plays an important role in Xinjiang’s economic growth and development. Due to the monoculture, cotton aphid has become a notorious problem. Foliar application of chemical pesticides is a common way of controlling aphid. However, foliar application of pesticides has negative effects on the environment and beneficial organisms, particularly in bees [15,16]. Bees are essential pollinators in natural ecosystems and agricultural crops [35,36]. The risk of pesticides to bees has become a worldwide concern and attracted increasing research on bee safety [36,37]. As a part of the efforts, this study investigated the residue levels of sulfoxaflor applied via drip irrigation in cotton pollen and nectar at different times and its potential risk to *A. mellifera*. Our results showed that the closer the application time to the flowering period was, the higher the concentration of sulfoxaflor in pollen and nectar of cotton could be. Because sulfoxaflor is a pesticide that can be easily degraded in cotton plants [38], the longer the time after application is, the lower the concentration of sulfoxaflor in pollen and nectar will be. Compared to the pesticide residues reported by Siviter et al. [23], concentrations of sulfoxaflor in pollen and nectar were lower in our study. This difference might result from different application methods. Because sulfoxaflor applied via foliar spray was directly on the surface of plants, which could result in flowers with a higher concentration of sulfoxaflor. On the other hand, sulfoxaflor applied through drip irrigation was not on the surface of plants, and honey bees could have no direct contact with the pesticide. Our results manifested that drip irrigation reduced the potential risk of sulfoxaflor to honey bees. Alarcón (2005) also reported that drip application of thiamethoxam reduced the side effect on bumble-bees [39]. By comparison, the concentrations of sulfoxaflor in pollen were higher than those in nectar. This trend concurred with references [23,28]. Additionally, there was no significant differences between 2016 and 2017 in sulfoxaflor concentrations in pollen and nectar of cotton. This could be due in part to the similar weather conditions where the average daily air temperature, mean monthly precipitation, and mean daily sunshine duration of the field sites were 24.2 °C, 81.3 mm, and 12.2 h in 2016 and 25.3 °C, 46.5 mm, and 13.4 h in 2017.

Bees can be exposed to insecticide in two ways in open fields: (1) Bees are directly exposed to drift droplets from foliar spray, or dust from seed drilling at planting, or inhalation of volatile pesticides during or after application and (2) bees are exposed to residues in pollen, nectar, honey, and water [40,41,42,43]. In this study, our risk assessment dealt only with residues in pollen and nectar. Because bees were mainly exposed to sulfoxaflor by pollen and nectar when sulfoxaflor was applied via drip irrigation [44]. Firstly, we assessed the contact exposure of sulfoxaflor on honey bees by the flower hazard quotient (FHQ_do_) [29], and the results showed that all FHQ_do_ values were lower than 0.1, indicating that sulfoxaflor residues in pollen and nectar were safe to honey bees ( Table 4; Table 5) [29]. Honey bees generally have different types, such as worker larvae, nurses, and forager. Different types of honey bees have different habits and different sensitivities to residues of pesticides [30]. Therefore, the dietary exposure of sulfoxaflor to three different types of honey bees (worker larvae, nurses, and foragers) was estimated by the T_50_, and the results showed that all T_50_ values were longer than the lifespan of three different types of honey bees [30], suggesting that residues of sulfoxaflor in pollen and nectar were safe to three different types of bees (Table 6; Table 7). In general, worker larvae and foragers of honey bees do not eat nectar, they eat honey. Nectar is dehydrated to concentrate sugar to honey. According to reference [33], honey contains an average of 80% of sugar. Meanwhile, the nectar of cotton flowers contains 17.9%−36.5% of sugar [34]. Therefore, the concentration of sulfoxaflor in nectar would be concentrated and the concentration of sulfoxaflor in honey was to be higher in honey. Our calculation showed that the concentrations of sulfoxaflor in honey could be within 46.1–96.4 μg·kg^−1^. According to the above results of risk assessment, the drip application of two different doses of sulfoxaflor before or during flowering appeared to have little negative risk to honeybees. Thus, drip irrigation could be an environmentally friend way of applying systemic insecticides for controlling aphid while safeguarding honeybees.

## 5. Conclusions

This is the first systematic evaluation of the safety of sulfoxaflor applied through drip irrigation at different times before flowering and during flowering to *A. mellifera*. Even though the drip application of high dose of sulfoxaflor during flowering resulted in higher residues in pollen and nectar, risk assessments by contact exposure and dietary exposure showed that drip application of sulfoxaflor should have little negative effects on honeybees. Our results indicate that the drip application of sulfoxaflor could represent a sustainable way of controlling aphid while protecting beneficial organisms during cotton production.

## Figures and Tables

**Table 1:** The calendar for conducting field experiments in Xinjiang, China in 2016 and 2017.

Date	Activity
4 April 2016	Sowing seeds
3 June 2016	Sulfoxaflor applied at 450 g a.i. ha^−1^ or 700 g a.i. ha^−1^ via drip irrigation 30 days before cotton flowering
13 June 2016	Sulfoxaflor applied at 450 g a.i. ha^−1^ or 700 g a.i. ha^−1^ via drip irrigation 20 days before cotton flowering
13 June 2016	Sulfoxaflor applied at 450 g a.i. ha^−1^ or 700 g a.i. ha^−1^ via drip irrigation 10 days before cotton flowering
7 July 2016	Sulfoxaflor applied at 450 g a.i. ha^−1^ or 700 g a.i. ha^−1^ via drip irrigation during cotton flowering
24 April 2017	Sowing seeds
10 June 2017	Sulfoxaflor applied at 450 g a.i. ha^−1^ or 700 g a.i. ha^−1^ via drip irrigation 30 days before cotton flowering
20 June 2017	Sulfoxaflor applied at 450 g a.i. ha^−1^ or 700 g a.i. ha^−1^ via drip irrigation 20 days before cotton flowering
30 June 2017	Sulfoxaflor applied at 450 g a.i. ha^−1^ or 700 g a.i. ha^−1^ via drip irrigation 10 days before cotton flowering
14 July 2017	Sulfoxaflor applied at 450 g a.i. ha^−1^ or 700 g a.i. ha^−1^ via drip irrigation during cotton flowering

**Table 2:** Concentration of sulfoxaflor in pollen and nectar of cotton plants applied with two doses of sulfoxaflor through drip irrigation at different times in 2016.

Application Time	Days after Treatment	Concentration (μg·kg^−1^) ± SE			
		450 g a.i. ha^−1^		700 g a.i. ha^−1^	
		Pollen	Nectar	Pollen	Nectar
30 days before flowering	35d	BDL ^z^	BDL ^y^	BDL	BDL
	40d	BDL	BDL	BDL	BDL
	45d	BDL	BDL	BDL	BDL
	50d	BDL	BDL	BDL	BDL
20 days before flowering	25d	BDL	BDL	8.9 ± 1.4c ^x^	BDL
	30d	BDL	BDL	BDL	BDL
	35d	BDL	BDL	BDL	BDL
	40d	BDL	BDL	BDL	BDL
10 days before flowering	15d	5.7 ± 1.5c	BDL	14.2 ± 1.4c	BDL
	20d	BDL	BDL	8.5 ± 1.6c	BDL
	25d	BDL	BDL	BDL	BDL
	30d	BDL	BDL	BDL	BDL
During flowering	0.08d	BDL	BDL	BDL	BDL
	1d	BDL	BDL	BDL	BDL
	3d	BDL	BDL	18.0 ± 1.3bc	BDL
	5d	17.0 ± 3.5c	BDL	39.2 ± 4.0a	13.8 ± 2.6c
	7d	15.3 ± 2.6c	BDL	31.7 ± 4.8ab	6.6 ± 1.3c
	15d	7.7 ± 2.3c	BDL	16.9 ± 4.3c	BDL
	20d	BDL	BDL	11.7 ± 2.9c	BDL

^z^ BDL (below detectable level) < 1.16 μg·kg^−1^. ^y^ BDL (below detectable level) < 0.68 μg·kg^−1^. ^x^ Means followed by different letters in the same column indicate significant differences in sulfoxaflor residue levels based on Tukey’s HSD test (*p* < 0.05).

**Table 3:** Concentration of sulfoxaflor in pollen and nectar of cotton plants applied with two doses of sulfoxaflor through drip irrigation at different times in 2017.

Application Time	Days after Treatment	Concentration (μg·kg^−1^) ± SE			
		450 g a.i. ha^−1^		700 g a.i. ha^−1^	
		Pollen	Nectar	Pollen	Nectar
30 days before flowering	35d	BDL ^z^	BDL ^y^	BDL	BDL
	40d	BDL	BDL	BDL	BDL
	45d	BDL	BDL	BDL	BDL
	50d	BDL	BDL	BDL	BDL
20 days before flowering	25d	BDL	BDL	6.6 ± 1.1de ^x^	BDL
	30d	BDL	BDL	BDL	BDL
	35d	BDL	BDL	BDL	BDL
	40d	BDL	BDL	BDL	BDL
10 days before flowering	15d	7.0 ± 1.8de	BDL	13.5 ± 1.9bcde	BDL
	20d	BDL	BDL	5.8 ± 1.6e	BDL
	25d	BDL	BDL	BDL	BDL
	30d	BDL	BDL	BDL	BDL
During flowering	0.08d	BDL	BDL	BDL	BDL
	1d	BDL	BDL	BDL	BDL
	3d	BDL	BDL	16.7 ± 1.3bcd	BDL
	5d	15.9 ± 1.2bcde	BDL	34.6 ± 4.9a	10.8 ± 1.1cde
	7d	10.9 ± 1.4cde	BDL	23.3 ± 1.7b	7.6 ± 1.9cde
	15d	BDL	BDL	17.5 ± 1.4bc	BDL
	20d	BDL	BDL	8.9 ± 1.5cde	BDL

^z^ BDL (below detectable level) < 1.16 μg·kg^−1^. ^y^ BDL (below detectable level) < 0.68 μg·kg^−1^. ^x^ Means followed by different letters in the same column indicate significant differences in sulfoxaflor residue levels based on Tukey’s HSD test (*p* < 0.05).

**Table 4:** Contact exposure risk levels of sulfoxaflor applied through drip irrigation at different times to *A. mellifera* in 2016.

Application Time	Days after Treatment	Contact Flower Hazard Quotient (FHQ_do_)	
		700 g a.i. ha^−1^	450 g a.i. ha^−1^
30 days before flowering	35d	-	-
	40d	-	-
	45d	-	-
	50d	-	-
20 days before flowering	25d	-	0.02
	30d	-	-
	35d	-	-
	40d	-	-
10 days before flowering	15d	0.01	0.02
	20d	-	0.01
	25d	-	-
	30d	-	-
During flowering	0.08d	-	-
	1d	-	-
	3d	-	0.03
	5d	0.03	0.09
	7d	0.03	0.07
	15d	0.01	0.03
	20d	-	0.02

**Table 5:** Contact exposure risk levels of sulfoxaflor applied through drip irrigation at different times to *A. mellifera* in 2017.

Application Time	Days after Treatment	Contact Flower Hazard Quotient (FHQ_do_)	
		450 g a.i. ha^−1^	700 g a.i. ha^−1^
30 days before flowering	35d	-	-
	40d	-	-
	45d	-	-
	50d	-	-
20 days before flowering	25d	-	0.01
	30d	-	-
	35d	-	-
	40d	-	-
10 days before flowering	15d	0.01	0.02
	20d	-	0.01
	25d	-	-
	30d	-	-
During flowering	0.08d	-	-
	1d	-	-
	3d	-	0.03
	5d	0.03	0.08
	7d	0.02	0.05
	15d	-	0.03
	20d	-	0.02

**Table 6:** Dietary exposure risk levels of sulfoxaflor to three different types of *A. mellifera* in 2016.

Application Time	Days after Treatment	T_50_^1^ (days)					
		450 g a.i. ha^−1^			700 g a.i. ha^−1^		
		Worker Larvae	Nurses	Forager	Worker Larvae	Nurses	Forager
20 days before flowering	25d	-	-	-	19,101.1	3232.5	-
10 days before flowering	15d	29,824.6	5047.2	-	11,971.8	2026.0	-
	20d	-	-	-	20,000.0	3384.6	-
During flowering	3d	-	-	-	9444.4	1598.3	-
	5d	10,000	1692.3	-	102.4–204.0	733.9	37.8–475.5
	7d	11,111.1	1880.3	-	210.7–413.1	907.6	79.1–994.2
	15d	22,077.9	3736.3	-	10,059.2	1702.3	-
	20d	-	-	-	14,529.9	2458.9	-

^1^ Time to reach oral LD_50_.

**Table 7:** Dietary exposure risk levels of sulfoxaflor to three different types of *A. mellifera* in 2017.

Application Time	Days after Treatment	T_50_^1^ (days)					
		450 g a.i. ha^−1^			700 g a.i. ha^−1^		
		Worker Larvae	Nurses	Forager	Worker Larvae	Nurses	Forager
20 days before flowering	25d	-	-	-	25,757.6	4359.0	-
10 days before flowering	15d	24,285.7	4109.9	-	12,592.6	2131.1	-
	20d	-	-	-	29,310.3	4960.2	-
During flowering	3d	-	-	-	10,179.6	1722.7	-
	5d	10,691.82	1809.4	-	130.5–259.1	831.5	48.3–607.5
	7d	15,596.33	2639.4	-	185.6–369.1	1234.7	68.7–863.3
	15d	-	-	-	9714.3	1644.0	-
	20d	-	-	-	19,101.1	3232.5	-

^1^ Time to reach oral LD_50_.
